# Artificial Intelligence and Panendoscopy—Automatic Detection of Clinically Relevant Lesions in Multibrand Device-Assisted Enteroscopy

**DOI:** 10.3390/cancers16010208

**Published:** 2024-01-01

**Authors:** Francisco Mendes, Miguel Mascarenhas, Tiago Ribeiro, João Afonso, Pedro Cardoso, Miguel Martins, Hélder Cardoso, Patrícia Andrade, João P. S. Ferreira, Miguel Mascarenhas Saraiva, Guilherme Macedo

**Affiliations:** 1 Alameda Professor Hernâni Monteiro, Department of Gastroenterology, São João University Hospital, 4200-427 Porto, Portugal; francisco.cnm@gmail.com (F.M.); tiagofcribeiro@outlook.com (T.R.); pedro.marilio.cardoso@chsj.min-saude.pt (P.C.); miguel.pedro96@gmail.com (M.M.); anapatriciarandrade@gmail.com (P.A.); guilhermemacedo59@gmail.com (G.M.); 2 WGO Gastroenterology and Hepatology Training Center, 4050-345 Porto, Portugal; 3 Faculty of Medicine, University of Porto, Alameda Professor Hernâni Monteiro, 4200-427 Porto, Portugal; 4 Department of Mechanical Engineering, Faculty of Engineering, University of Porto, Rua Dr. Roberto Frias, 4200-465 Porto, Portugal; j.ferreira@fe.up.pt; 5 DigestAID—Digestive Artificial Intelligence Development, R. Alfredo Allen n°. 455/461, 4200-135 Porto, Portugal; 6 ManopH Gastroenterology Clinic, R. de Sá da Bandeira 752, 4000-432 Porto, Portugal; miguelms.manoph@gmail.com

**Keywords:** artificial intelligence, deep learning, panendoscopy, device-assisted enteroscopy

## Abstract

**Simple Summary:**

Device-assisted enteroscopy is the only diagnostic and therapeutic exam capable of exploring the entire gastrointestinal tract. However, the diagnostic yield of this procedure is not sufficient enough to assure a cost-effective panendoscopy, and there is significant interobserver variability during the exam. Artificial intelligence tools have been proved to be beneficial in several areas of medicine, namely in Gastroenterology, with a strong image component. However, the development of deep learning models for application in device-assisted enteroscopy is still in an embryonic phase. The authors herein aimed to develop a multidevice convolutional neural network based on 338 exams performed in two renowned centers. The present model was able to accurately identify multiple clinically relevant lesions across the entire gastrointestinal tract, with an image processing time that favors its clinical applicability. The first worldwide panendoscopic model showed the potential of artificial intelligence in augmenting the accuracy and cost-effectiveness of device-assisted enteroscopy.

**Abstract:**

Device-assisted enteroscopy (DAE) is capable of evaluating the entire gastrointestinal tract, identifying multiple lesions. Nevertheless, DAE’s diagnostic yield is suboptimal. Convolutional neural networks (CNN) are multi-layer architecture artificial intelligence models suitable for image analysis, but there is a lack of studies about their application in DAE. Our group aimed to develop a multidevice CNN for panendoscopic detection of clinically relevant lesions during DAE. In total, 338 exams performed in two specialized centers were retrospectively evaluated, with 152 single-balloon enteroscopies (Fujifilm®, Porto, Portugal), 172 double-balloon enteroscopies (Olympus^®^, Porto, Portugal) and 14 motorized spiral enteroscopies (Olympus^®^, Porto, Portugal); then, 40,655 images were divided in a training dataset (90% of the images, *n* = 36,599) and testing dataset (10% of the images, *n* = 4066) used to evaluate the model. The CNN’s output was compared to an expert consensus classification. The model was evaluated by its sensitivity, specificity, positive (PPV) and negative predictive values (NPV), accuracy and area under the precision recall curve (AUC-PR). The CNN had an 88.9% sensitivity, 98.9% specificity, 95.8% PPV, 97.1% NPV, 96.8% accuracy and an AUC-PR of 0.97. Our group developed the first multidevice CNN for panendoscopic detection of clinically relevant lesions during DAE. The development of accurate deep learning models is of utmost importance for increasing the diagnostic yield of DAE-based panendoscopy.

## 1. Introduction

Device-assisted enteroscopy (DAE) is an exam that combines diagnostic properties with tissue sampling and therapeutic endoscopy. Initially conceived for the investigation of small bowels [1], DAE’s properties make it suitable for evaluation of the entire GI tract. Typically, DAE is performed with a single- or double-balloon enteroscope, but recently, the motorized spiral enteroscope has been an alternative [2].

DAE is commonly performed in various clinical settings, more commonly after capsule endoscopy (CE) findings [3]. Ulcers and erosions are the most common pathological findings in the small bowel, with diverse etiologies (namely Crohn’s disease, refractory celiac disease, infections and neoplasms) [4]. DAE is capable of exploring a greater length of ileal mucosa than conventional ileocolonoscopy, augmenting the diagnostic accuracy for small-bowel Crohn’s disease. Additionally, in the presence of stricturing Crohn’s disease, DAE allows balloon dilation of small-bowel strictures, reducing the need for surgery.

Additionally, the identification of small-bowel tumors in CE is a common indication for DAE, with the possibility of tissue sampling being crucial to disease management [5,6]. Moreover, DAE is a useful exam in the management of polyposis syndromes, namely Peutz–Jeghers syndrome, with endoscopic polypectomy as an alternative to small-bowel resection in the course of multiple lesions found during the disease’s course [7]. Finally, DAE allows marking of the area by a small-bowel neoplasia, enhancing minimally invasive surgery.

Additionally, DAE is commonly performed in the setting of obscure gastrointestinal bleeding, particularly after a positive CE exam [8]. Angioectasias are the most common finding in the setting of small-bowel bleeding, with the possibility of argon plasma coagulation during the exam after lesion detection. However, the diagnostic yield in the presence of OGIB is modest (56%, increasing to 75% if with a positive CE exam) [8].

Additionally, there has been an increased focus on double-balloon-assisted colonoscopy. In fact, the use of a double-balloon enteroscope is useful in technically difficult colonoscopies, achieving higher cecal intubation cases and less patient discomfort in patients with difficult or previous incomplete colonoscopies [9]. However, this alternative use of the enteroscope should be accompanied by a high diagnostic accuracy. Finally, DAE is also performed in settings of altered anatomy. One of the main examples is the performance of DAE in order to access an excluded stomach in patients with Roux-en-Y gastric bypass in the setting of gastrointestinal bleeding or malignancy suspicion [10]. These alternative uses of the enteroscope guide the need to enhance the diagnostic accuracy of DAE, not only in the small bowel evaluation but also in a panendoscopic setting. Thus, despite the potential capabilities of DAE, there is a need to increase its diagnostic yield in several clinical settings.

In recent years, the application of artificial intelligence (AI) technologies in the medical field has observed exponential growth. Convolutional neural networks (CNNs) are a human cortex-inspired multi-layer architecture, with high proficiency for image pattern detection [11]. As a matter of fact, CNN models have been developed in several medical areas [12,13,14]. CE has been a focus of study in the development and application of deep learning technologies, increasing its diagnostic yield with a significant reading time reduction [15,16,17]. In fact, the development of deep learning methods has been theorized as a potential revolutionary tool to increase the diagnostic accuracy and cost-effectiveness of DAE [10]. Nevertheless, the implementation of AI models for DAE is still in the early stages. In fact, AI application in DAE has been studied for the identification of vascular lesions, protuberant lesions, ulcers and erosions [18,19,20]. Nevertheless, the clinical application of such technology is dependent on the ability to identify different types of lesions throughout a complete exam, while functioning in different devices. In this study, our group aimed to develop the first worldwide multibrand CNN for panendoscopic automatic classification of clinically relevant lesions in DAE, namely, vascular lesions, hematic residues, protuberant lesions, ulcers and erosions.

## 2. Materials and Methods

### 2.1. Study Design

A bicentric study was performed for construction of the CNN. A total of 338 exams consecutively performed at two Portuguese specialized centers (Centro Hospitalar Universitário São João and ManopH) between January 2020 and May 2023 were used for the development of the CNN. During that period, DAE was performed by three experienced gastroenterologists using three different devices: the double-balloon enteroscopy system Fujifilm EN-580T (*n* = 226), the single-balloon enteroscopy system Olympus EVIS EXERA II SIF-Q180 (*n* = 98) and the Olympus PowerSpiral Motorized Enteroscope PSF-1 (*n* = 14). Our study respected the Declaration of Helsinki and was developed in a non-interventional fashion. The study was approved by the ethics committee of São João University Hospital/Faculty of Medicine of the University of Porto (No. CE 407/2020). Omission of potentially identifying information of the subjects was ensured and each patient received a random number assignment to obtain effective data anonymization for researchers involved in the CNN. A legal team with Data Protection Officer (DPO) certification was responsible for the non-traceability of the data in conformity with general data protection regulation (GDPR).

### 2.2. Lesion Classification

The CNN comprised enteroscopy images from esophagic, gastric, enteric and colonic segments. Each segment was reviewed in order to identify several categories of lesions. The lesions selected for the model’s training and evaluation comprised a group of clinically relevant alterations in the gastrointestinal mucosa, in which the CNN could have a role with diagnostic and therapeutic implications. Vascular lesions included red spots, angioectasia and varices. Red spots were considered as punctuate flat lesions with a diameter under 1 mm, without vessel appearance. Angioectasia were defined as a reddish lesion of tortuous dilated clustered capillaries. Otherwise, varices were defined as venous dilations with a serpiginous appearance. The protruding lesions were defined as tissue elevations above the gastrointestinal epithelium, including polyps, flat lesions, subepithelial lesions and nodules. Otherwise, ulcers were considered whitish base areas of loss of epithelial covering, with surrounding swollen mucosa and a diameter of at least 5 mm. Mucosal erosions consisted of areas of minimal loss of epithelial covering and normal surrounding mucosa. The extracted images were classified by gastroenterologists with expertise in DAE (MMS, HC, PA). Non-agreeable images were discussed between DAE experts and discarded in the absence of a consensus.

### 2.3. CNN Development

**Figure 1:**
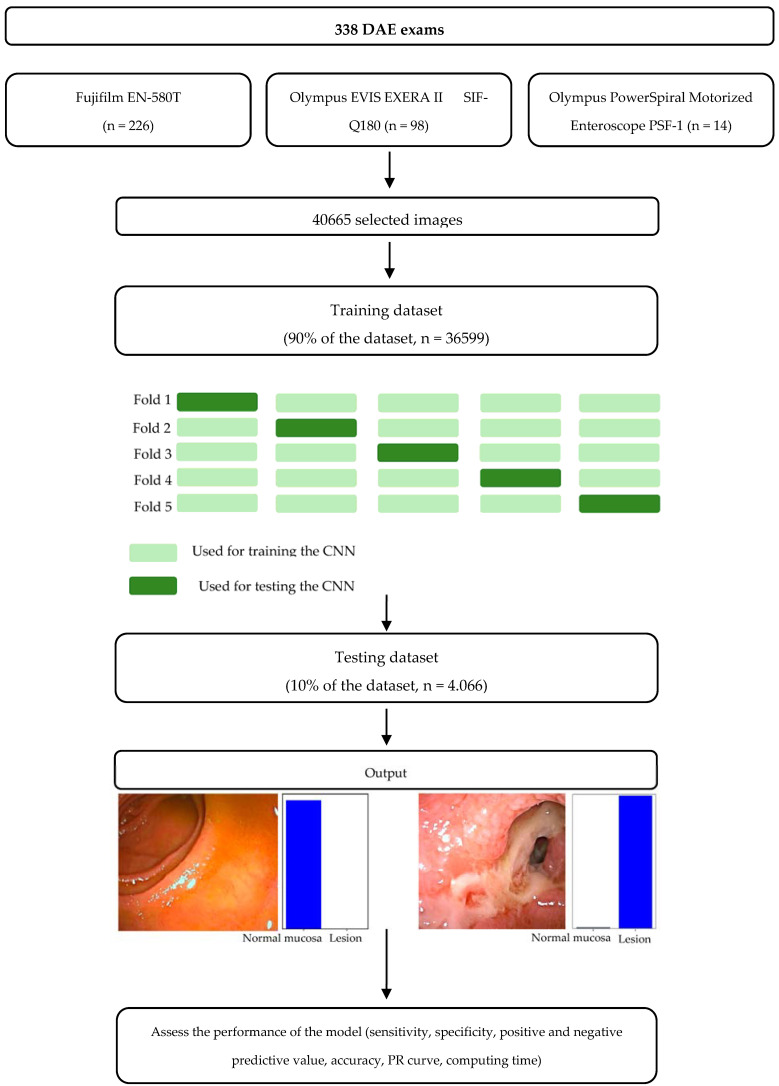
Study flow chart for the training and testing phases. DAE—Device-assisted enteroscopy. The term lesion refers to the presence of clinically relevant lesions.

The study design is represented through a flowchart in Figure 1. A total of 40,665 images were used for developing the model. The full dataset was divided into a training dataset (comprising around 90% of the images, *n* = 36,599) and a testing dataset (comprising around 10% of the images, *n* = 4066). The testing dataset was used for evaluating the model. In the training dataset, a 5-fold cross validation was performed, dividing the training dataset into 5 similar sized subsets. The results of each subset were used to identify the best parameters of the model that were used in the testing dataset.

The CNN was created with the Xception model pre-trained on ImageNet. The convolutional layers of the model were kept, assuring the transference of the learning to our data, while the last fully connected layers were removed. The attachment of fully connected layers was based on the number of the classes for classification of DAE images.

The model had 2 blocks with fully connected layers followed by a Dropout layer of 0.25 drop rate. A Dense layer with a size based on the number of categories to classify was added. A learning rate of 0.0001, batch size of 64 and number of epochs of 20 was set by trial and error. Our group used Keras libraries and Tensor-flow 2.3 to prepare the data and run the model. The analysis was dependent on a computer with an Intel^®^ Xeon^®^ Gold 6130 processor (Intel, Santa Clara, CA, USA) and a NVIDIA Quadro^®^ RTXTM 4000 graphic processing unit (NVIDIA Corporate, Santa Clara, CA, USA).

### 2.4. Performance Measures and Statistical Analysis

**Figure 2:**
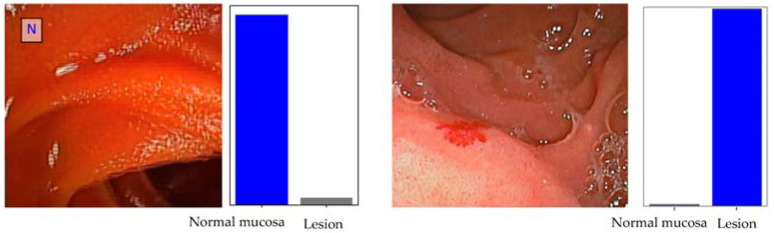
Output obtained from the convolutional neural network. The bars are a representation of the estimated probability by the CNN. The model output was given by the finding with the highest probability. The blue bars represent a correct prediction, whereas incorrect predictions are represented by grey bars.

**Figure 3:**
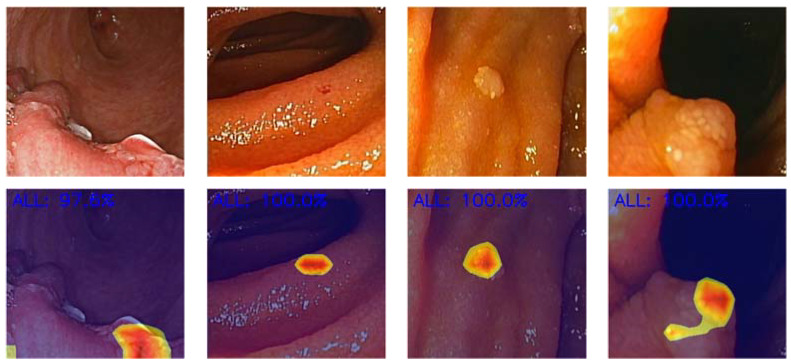
Heatmaps generated by the convolutional neural network selecting the image location responsible for the identification of clinically relevant panendoscopic lesions. The given probability represents the level of certainty in lesion prediction.

The binary CNN calculated the probability of normal mucosa versus clinically relevant lesions for each given image (Figure 2), with higher probabilities demonstrating greater CNN prediction confidence. Heatmaps were generated based on localized features responsible for the prediction of the model (Figure 3), attempting to achieve a better comprehension of the model and guide clinical decision either during tissue sampling or therapeutic procedures. The CNN’s classification was compared to three DAE experts’, which remains the gold standard for evaluation of DAE images. Table 1 translates the confusion matrix between experts and CNN classification.

The model was evaluated through its sensitivity, specificity, positive predictive value (PPV), negative predictive value (NPV) and accuracy (Table 2). These performance measures were represented with their means and 95% confidence intervals (CI). The model’s global performance was evaluated with the precision recall (PR) curve and area under the precision recall curve (AUC-PR). Sci-kit learn version 0.22.2 was used for the statistical analysis [21].

## 3. Results

### 3.1. Construction of the Network

A CNN model was constructed with 40,655 images from 338 DAE exams. The training dataset, with 90% of the total images, was split into five similar-sized independent subsets. The remaining 10% of the total images were used for the validation dataset.

The CNN evaluated each individual image, predicting a classification with a level of certainty, later compared with the expert’s classification. The inputs of the different subsets of the training dataset allowed the trimming of the individual parameters of the CNN, which were evaluated in the validation dataset.

### 3.2. Global Performance of the Network

The training dataset was developed with a five-fold cross validation. Table 2 demonstrates the performance results of the folds of the training model. Thus, the training dataset had 88.7% mean sensitivity, 98.0% specificity, 92.6% PPV and 97.0% NPV, with a mean accuracy of 96.0%.

The validation dataset, with the remaining 10% of the total images, was used for evaluation of the CNN’s performance. Table 1 shows the confusion matrix between the CNN’s prediction and the experts’ classification. The deep learning model had a sensitivity of 88.9%, a specificity of 98.9%, a PPV of 95.8% and an NPV of 97.1%. The model revealed an overall accuracy of 96.8% and an AUC-PR of 0.97.

### 3.3. Convolutional Neural Network Computational Performance

The CNN completed the evaluation of the testing dataset within 33 s, resulting in an image processing time of 124 images per second.

## 4. Discussion

In this multicentric study, our group developed a CNN capable of automatic identification of clinically relevant lesions in a panendoscopic setting during DAE. Our model revealed excellent performances in all the evaluated parameters, with an overall accuracy of 96.8%. These results were accompanied by an image processing time that favors the clinical applicability of the technology. Additionally, the CNN was developed in a multidevice setting, namely, single-balloon enteroscope, double-balloon enteroscope and motorized spiral enteroscope devices, including all the types of devices used during DAE. This is, to our knowledge, the first worldwide multidevice CNN capable of detecting several types of lesions in esophagic, gastric, enteric and colonic segments during DAE.

Furthermore, it is important to discuss some methodologic points of our study. The CNN was trained with a five-fold cross validation strategy. This design assures a balanced distribution of different classes between folds, which is important in cases where class imbalance is common [22]. The choice of a five-fold cross validation strategy significantly reduced the random fluctuation typical of a single training–testing split. This methodological point aids in creating a model capable of better generalization of unseen data—which is of utmost importance in medical technology development and application [23]. Additionally, our group opted for the use of PR curves instead of the commoner receiver operating characteristic (ROC) curves to evaluate the discriminating ability of the model. The current literature regards the excessive optimism of ROC curves in evaluating a model performance in cases of data imbalance [24,25]. In cases of data imbalance, PR curves are more informative and preferred [26]. Thus, in this CNN, normal images corresponded to around 80% of the total images, favoring the use of PR curves, taking into consideration the objective of identifying all the lesions images, instead of the commoner normal images (which are part of the ROC concept).

On the other hand, beyond the increased complexity in terms of model characteristics, a CNN should be capable of being trustworthy and comprehensible. Thus, in the last few years, the concept of explainable AI has been a matter of intense discussion [27,28]. Our group tried to address this need with the development of heatmaps for each classified image, identifying the area responsible for the classification of the image (normal mucosa vs. lesion). Therefore, our group recognizes that the development of heatmaps and other explainable AI methods is of great interest to address the performance of procedures during DAE (like tissue sample or argon plasma coagulation of angioectasias), but also to ensure trustworthiness of the model and confidence among clinicians, who are responsible for its use and implementation. Thus, addressing this question is of utmost importance not only for the model’s developers but also for regulatory entities, ensuring accountability during the AI development and implementation process.

In addition to explainability, data responsibility and the ethical or legal consequences of the use of AI models are a matter of great interest [29]. Firstly, there is a need to discuss the legal responsibility in the face of an adverse outcome or incorrect diagnosis. There is still difficulty in establishing the autonomy and responsibility of an artificial intelligence model. In fact, in case of an adverse event, it is important to determine if any of the parts failed and determine responsibilities. Nevertheless, it is possible to have a misdiagnosis in the absence of a clearly determined error from any part. On the other hand, a clinician that assumes a decision based uniquely on a model’s output may be less trustworthy by the patient [30]. Therefore, it is important to have deep learning models’ outputs critically analyzed by experts in the field. Additionally, the development and implementation of deep learning models is commonly slowed by clinicians’ fear to be replaced by AI tools. Contrarily, AI models should be interpreted as a tool to increase clinicians’ diagnostic accuracy and provide more time for patient care. Nevertheless, perfect synergy is highly dependent on two factors: avoidance of the loss of skill by the clinician and a clear definition of decision protocol in cases of machine–human disagreement [31]. The authors have addressed these questions in the rewritten Discussion section of the paper.

The implementation of AI-based technologies in medicine is highly dependent on its generalization for use in multiple devices [32]. The FAIR data principles were published in 2016 as guiding tools for research data stewardship [33]. In order to fulfill the FAIR principles, data should be findable, accessible, interoperable and reusable. In the present study, the data are findable as they are assigned with unique and confidential classifiers and saved in our records. Moreover, they also respect the principle of accessibility as they can be accessed by the study investigators respecting the patient confidentiality issues and ethical principles described in the methods section. Additionally, our data are reusable, conceived to allow the continuous development of the convolutional neural network and application in another models. Taking into account the need to fulfill these four principles, our group regards the proof of methodological development in the fulfillment of system interoperability, which is not always addressed by the majority of deep learning models published in Gastroenterology. Thus, the interoperability challenge is a discussion topic in multiple science fields [34,35,36]. Our group has addressed that concern, developing a CNN that works in three different enteroscopes, comprising single-balloon, double-balloon and motorized spiral enteroscopy devices, solving a fundamental interoperability challenge for applying the technology. This is, to our knowledge, the first worldwide CNN for panendoscopic detection of clinically relevant lesions in a multidevice setting, achieved through a large image dataset.

The development of artificial intelligence models encompasses several methodological steps, translated by the technological readiness level (TRL) scale [37]. In fact, the TRL is very different between Gastroenterology fields. For instance, capsule endoscopy is one of the main areas for the development of deep learning models in Gastroenterology [38,39]. However, the majority of studies are still in an early development phase and not validated in the clinical practice. On the other hand, in colonoscopy, a computer-aided diagnostic software is already disposable in clinical practice (GI Genius, Medtronic^®^), being proficient in detecting colorectal polyps and predicting their histology [40]. Additionally, the software is capable of working with different endoscopy devices, achieving a solution for the interoperability challenge. However, the technology is dedicated to the evaluation of colonic mucosa, unsuitable for a panendoscopic evaluation of the gastrointestinal tract, and is centered in the detection of protuberant lesions. Our model, although being developed in a less-performed exam like device-assisted enteroscopy, focuses not only on the achievement of system interoperability but also the panendoscopic detection of multiple clinically relevant lesions. However, it is still at a low TRL and needs larger prospective real-time studies to implement the model into clinical practice.

DAE is a safe procedure, albeit with non-neglectable risks for adverse events, which are mostly minor and self-limiting. The advent of spiral motorized enteroscopy was accompanied by an increase in these minor adverse events. In fact, a work by Singh et al. revealed a minor adverse events rate of 48%, including superficial mucosal tears with middle ooze and mucosal tears [41]. Additionally, the distinction between these minor iatrogenic lesions and true lesions is a challenge in DAE. Differentiating angioectasias from iatrogenic lesions or red spots is challenging. Additionally, protuberant lesions like those verified in Peutz–Jeghers syndrome can be mistaken with irrelevant xanthelasmas. The impossibility to distinguish between these entities can prompt to unnecessary treatment, with non-neglectable risks of complications or an increase in procedure time. Deep learning models have been proposed to have a role in these clinical settings, with the possibility of not only increasing the diagnostic accuracy of an exam but also reducing the rate of unnecessary procedures. Thus, our model addressed this question, revealing 98.0% specificity with great accuracy for distinguishing between vascular lesions, ulcers, erosions and minor iatrogenic lesions developed during the exam, especially when performing spiral enteroscopy. This technical specificity is of great interest in reducing the performance of unnecessary argon plasma coagulation procedures, which are not only iatrogenic but also require higher exam completion time and are associated with increased exam-related costs.

The concept of panendoscopic evaluation of the GI tract with a single exam was introduced with the advent of minimally invasive capsule panendoscopy [42,43]. Nevertheless, capsule endoscopy (CE) is incapable of tissue sampling and therapeutic procedures. Therefore, DAE is the only exam with therapeutic purposes capable of the evaluation and management of esophageal, gastric, enteric and colonic mucosa pathologies. Thus, the development of a CNN capable of identifying lesions in a panendoscopic setting is of utmost importance to increase the clinical applicability of the model and the exam itself. Indeed, this is the first validated model to detect several clinically relevant lesions in a panendoscopic setting, achieving excellent results throughout esophagus, stomach, enteric and colonic segments of the exam. This model represents a critical milestone in implementing AI in digestive endoscopy, increasing the cost effectiveness of the exam in a panendoscopic evaluation.

In spite of the exponential growth in the development of deep learning models for CE [44,45], the application of AI technologies to DAE is still in a premature state, with scarce works applying deep learning models to augment the diagnostic performance of the exam. Additionally, the existing works were focused on detecting a specific type of lesion [18,19,20], which guarantees a diminished clinical applicability and a lower technology readiness level (TRL) of the technology. This work constitutes a landmark with the development of a CNN capable of detecting clinically relevant lesions during DAE, namely, vascular and protuberant lesions, hematic residues, ulcers and erosions. Additionally, this work was developed with images of both single-balloon, double-balloon and motorized spiral enteroscopes, solving a relevant interoperability challenge. The development of an explainable AI method like heatmap generation is of great importance to address the model’s trustworthiness and accountability.

Our group trained and validated the first panendoscopic CNN for detecting clinically relevant lesions during DAE, with high global accuracy and image processing capacities. Nevertheless, our CNN is still in an embryonic phase and not ready for clinical applicability.

This study contains some limitations. Firstly, it was developed with a retrospective design. Therefore, there is a need for larger prospective multicentric studies in the future to ensure clinical implementation of the models. Secondly, the CNN was based on still images, creating a need for real-time evaluation of panendoscopic lesions during DAE.

## 5. Conclusions

In conclusion, DAE is the only exam with diagnostic and therapeutic purposes capable of assuring panendoscopic evaluation of GI tract. However, the multiplicity of findings during this exam and regional differences between the mucosa of the different GI tract portions favors the implementation of AI models to increase the diagnostic ability and aid during therapeutic procedures. To our knowledge, our study reveals the first deep learning model capable of identifying clinically relevant lesions in a panendoscopic and multidevice setting (including the majority of DAE devices used in clinical practice). The development and application of these systems could amplify the indications and benefits of DAE, increasing its diagnostic yield and cost-effectiveness. In the future, larger prospective multicentric studies are needed to develop and apply these models.

## Figures and Tables

**Table 1:** Confusion matrix of automatic detection versus the expert’s classification in the testing dataset of the CNN model. Number of cases (relative frequency).

		Experts Classification	
		Normal Mucosa	Clinically Relevant Lesions
CNN Classification	Normal mucosa	3168 (0.97)	96 (0.03)
	Clinically relevant lesions	34 (0.04)	769 (0.96)

**Table 2:** Performance measures of the 5-fold cross validation of the training dataset and testing dataset for panendoscopic detection of clinically relevant lesions. N—number of patients. Sn—sensitivity. Sp—specificity. PPV—positive predictive value. NPV—negative predictive value. Acc—accuracy. ()—95% confidence interval values.

	Sn	Sp	PPV	NPV	Acc
Fold 1	0.87	0.95	0.81	0.96	0.93
Fold 2	0.87	0.97	0.90	0.96	0.95
Fold 3	0.89	0.99	0.97	0.97	0.97
Fold 4	0.91	0.99	0.97	0.98	0.97
Fold 5	0.90	0.99	0.98	0.97	0.97
Training dataset mean N = 38,599	0.887 (0.880–0.895)	0.980 (0.978–0.981)	0.926 (0.920–0.931)	0.970 (0.968–0.972)	0.960 (0.958–0.962)
Testing dataset N = 4068	0.889 (0.866–0.909)	0.989 (0.985–0.993)	0.958 (0.942–0.969)	0.971 (0.965–0.976)	0.968 (0.962–0.973)

## Data Availability

The data presented in this study are available in this article.
